# SOX11 is a biomarker for cyclin D1-negative mantle cell lymphoma

**DOI:** 10.1186/s40364-016-0060-9

**Published:** 2016-03-03

**Authors:** Roshni Narurkar, Mohammad Alkayem, Delong Liu

**Affiliations:** Department of Medicine, Westchester Medical Center and New York Medical College, Valhalla, NY 10595 USA

## Abstract

Cyclin D1 (CCND1) protein overexpression and/or the t(11;14)(q13;q32) translocation are the pathognomonic hallmarks of mantle cell lymphoma (MCL). However, there have been cases that lacked both t(11;14) and cyclin D1 protein but still had a gene expression profile suggesting a diagnosis of MCL. SOX11 expression was detected in most cyclin D1- negative MCL and can serve as a specific biomarker for the diagnosis of this subset of MCL. Lack of SOX11 expression in MCL was associated with an indolent subset and favorable prognosis.

## Background

Mantle cell lymphoma (MCL) is a distinct subtype of non-Hodgkin lymphoma [1]. The immunophenotype of the cells resembles the lymphocytes in the mantle zone of normal germinal follicles, and is characterized by co-expression of B-cell antigens (CD19+, CD20+, CD22+, CD43+, CD79+, surface immunoglobulin sIgM+, sIgD+) and the T-cell associated marker CD5+. MCL cells stain strongly for the antiapoptotic protein BCL-2, but are negative for germinal center markers like CD10 and BCL-6 [2, 3]. MCL has pathognomonic chromosomal translocation t(11;14), leading to constitutive cyclin D1 overexpression. Cyclin D1 (CCND1) overexpression has been detected in 90 % of MCL patients [3]. SOX11 has been found to be specifically expressed in more than 90 % of MCL cases. It is especially interesting that SOX11 was found to be 100 % positive in MCL with negative CCND1/t(11,14) [4]. Recent literature describes an indolent form of MCL which has longer duration of survival [5, 6]. Indolent MCL typically has non-nodal presentation, hypermutated IGVH, lack of genomic complexity, and absence of SOX11 expression. Therefore, SOX11 can serve as a biomarker for diagnosis and prognosis of a subset of MCL.

### SOX11

SOX11 is an intronless gene which encodes a member of the SOX (SRY-related HMG-box) family of transcription factors. The gene has 8,719 bases, localized on chromosome 2p25.3 [7, 8]. SOX11 protein has 441 amino acid residues. SOX11 protein serves as a transcription factor with a DNA-binding high-mobility group (HMG) domain [9, 10].

SOX11 is a neural transcription factor for the developing central nervous system in the embryo and is involved in neurogenesis, neural cell survival and neurite outgrowth. It is also involved in the determination of cell fate, such as sex differentiation, skeletogenesis, and stemness.

SOX11 mutations and overexpression have been associated with autosomal dominant mental retardation, coffin-siris syndrome, mantle cell lymphoma, and malignant glioma. Sox11 mRNA is significantly more expressed in gliomas compared with healthy brain tissue, suggesting that SOX11 might play a role in malignant transformation and probably cell survival [11].

SOX11 C1 monoclonal antibody was used to detect the SOX11 expression in MCL using immunofluorescence and flow cytometry studies. The gene was detected in all MCL cases studied, including CCND1 negative MCL cases. SOX11 was not detected in one case of MCL which was diagnosed with t(11,14) and immunophenotyping. Such data prove that SOX11 is a useful diagnostic tool for MCL, irrespective of the CCND1 presence. It can also be detected by flow cytometry. SOX11 expression can help distinguish MCL from other B cell lymphomas like chronic lymphoid leukemia (CLL) as most of them do not stain for SOX11 [12, 13]. However, SOX11 expression detected by immunohistochemistry can be limited by the monoclonal antibodies used. A more specific antibody, MRQ-58, was shown to be able to overcome the problem [14].

### SOX11 as a biomarker for cyclin D1-negative MCL

It remains elusive for definitive diagnosis of cyclin D1- negative MCL. In a study of 238 mature B-cell neoplasms, the microarray database was re-examined for SOX11 expression. The samples underwent immunohistochemistry staining for SOX11 protein. There were 12 cases of cyclin D1-negative MCL, 54 cases of conventional MCL, and 209 other lymphoid neoplasms. SOX11 nuclear protein was positive in 50 cases (93 %) of conventional MCL and also in all the 12 cases (100 %) of cyclin D1-negative cases of MCL [4]. The SOX11 was also detected in the six cases of lymphoblastic lymphomas, in two of eight cases of Burkitt’s lymphoma, and in two of three T-prolymphocytic leukemias, but SOX11 expression was not detected in the remaining lymphoid neoplasms. It appears therefore that SOX11 expression is a highly specific biomarker for cyclin D1- negative mantle cell lymphoma.

Sensitive and specific RT-qPCR assay was reported and used in ongoing longitudinal study to evaluate minimal residual disease (MRD) in patients with MCL to display the correlation between treatment response and load of MRD [3]. One study investigated 69 cases of lymphomas, including MCL, marginal zone lymphoma, follicular lymphoma, small lymphocytic lymphoma, Burkitt lymphoma, plasma cell myeloma and benign lymph nodes. All of these samples underwent immunostains for SOX11. 100 % of the 13 cases of MCL was positive for SOX11 and the rest of the cases were negative [15]. These data suggested that SOX11 can serve as the genetic signature of most MCL [10, 16, 17].

### SOX11 as a biomarker for the prognosis in MCL

It has been reported that SOX11 helps not only in diagnosing cyclin D1 negative cases, but also helps distinguish indolent SOX11 negative MCL from classical MCL [18]. Indolent MCL has several genes under-expressed compared to aggressive classical MCL: SOX11 being one of them [5, 6].

In contrast to follicular lymphoma, small lymphocytic lymphoma and reactive lymphoid tissue, most MCLs (48/53 patients) in a study expressed SOX11 protein in the nucleus [19]. This nuclear expression pattern of SOX11 can therefore serve as a new biomarker for a subset of MCL. In this study, SOX11 was found to be only expressed in the cytoplasm in 5/53 MCL cases. These 5 MCL patients had a shorter survival compared to the 48 MCL patients with nuclear SOX11 expression. Hence, the SOX11 expression pattern in MCL may have a prognostic role.

IN another genomic and gene expression profiling analysis of MCL case series, 112 cases of MCL were analyzed for SOX11 expression [6]. Among these 112 cases, 15 cases were negative for SOX11 expression, and were found to be consistent with indolent MCL which had significantly better 5 year survival that those positive for SOX11 expression (78 % SOX11^-^ vs 36 % SOX11^+^, *p* = 0.001).

However, in a population based study of 186 cases of MCL, 13 cases lacked nuclear expression of SOX11, and had shorter overall survival. Therefore, SOX11 nuclear expression may be a predictive prognostic biomarker for indolent MCL in this study [20].

### SOX11 in other B cell malignancies

Another study surveyed SOX 11 expression in different forms of lymphoid malignancies. The study tested one hundred and seventy-two specimens by immunostaining for the SOX11 N and C termini. This series reported that nuclear SOX11 was strongly positive in most B and T-lymphoblastic leukemia/lymphomas and half of childhood Burkitt’s lymphomas, but only weak SOX11 expression was seen in hairy cell leukemia. SOX11 was negative in chronic lymphocytic leukemia/lymphoma, marginal zone, follicular and diffuse large B-cell lymphomas, as well as in cases of myeloma, Hodgkin’s lymphomas and mature T-cell and NK/T-cell lymphomas [13].

In a recent study of 60 MCL cases, cyclin D1 positive monotypic plasma cells and lymphoid cells with plasmacytic differentiation were observed in 7 SOX11-negative but in none of 41 SOX11-positive MCLs (*P* < 0.001) [21]. There have been suggestions that the presence of hypermutated immunoglobulin heavy chain variable gene (IGVH) in MCL has been associated with negative or low expression of SOX11. In a study of MCL specimens using global gene expression, IGVH-mutated/SOX11-negative MCL cases were shown to have a profile enriched in memory B cells-related signatures [22]. Therefore, these studies suggest that terminal B cell differentiation in MCL may be associated with SOX11 negative phenotypes.

## Conclusion

SOX11 expression was detected in most cyclin D1- negative MCL and can serve as a specific biomarker for the diagnosis of this subset of MCL. Lack of SOX11 expression was associated with indolent MCL and favorable prognosis.
